# Deep Vein Thrombosis among Intensive Care Unit Patients; an Epidemiologic Study

**Published:** 2017-01-09

**Authors:** MirMohammad Miri, Reza Goharani, Mohammad Sistanizad

**Affiliations:** 1 Department of Critical Care, Imam Hossein Medical and Educational Center, Shahid Beheshti University of Medical Sciences, Tehran, Iran.; 2 Department of Clinical Pharmacy, Faculty of Pharmacy, Shahid Beheshti University of Medical Sciences, Tehran, Iran.

**Keywords:** APACHE, ultrasonography, Doppler, venous thrombosis, intensive care units

## Abstract

**Introduction:**

Deep vein thrombosis (DVT) is a major cause of morbidity and mortality in intensive care unit (ICU) patients despite use of prophylactic anticoagulant therapy. The aim of the present study was to determine the incidence of DVT among medical and surgical ICU patients.

**Methods:**

In this cross sectional study, patients older than 18 years who were hospitalized in the ICU of Imam Hossein educational Hospital, Tehran, Iran, for ≥ 2 days, during August 2008 to July 2011 were evaluated regarding DVT incidence. Demographic data, comorbidities, acute physiology and chronic health evaluation (APACHE) II scores, ICU length of stay, type of DVT prophylaxis, and patient outcomes were analyzed using SPSS 19.

**Results:**

Out of the 1387 reviewed patient files, 500 (36.04%) patients had been classified as potential DVT cases. DVT occurred in 3.5% of them with the mean age of 60 ± 18 years (62.5% male) and mortality rate of 27.1%. Significant independent risk factors of DVT incidence were age (p = 0.02) and length of ICU stay (p = 0.01).

**Conclusion:**

The results of this study showed the 3.5% incidence of DVT in ICU admitted patients. Longer ICU stay and older age were independent risk factors of DVT development.

## Introduction

Venous thromboembolism (VTE) is the third most common cardiovascular disorder after myocardial infarction and stroke (1). The rate of morbidity and mortality associated with thromboembolic events is high, with 28-day fatality rates reported to be 9% for deep vein thrombosis (DVT) and 15% for pulmonary embolism (PE) (2). The causes of DVT may be acquired, inherited or a combination of both (3). The diagnosis and treatment of DVT are challenging and expensive (3). 

DVT can complicate the course of a disease, but may also be encountered in the absence of precipitating factors. While long-term morbidity due to post-thrombotic syndrome is common, and can be substantial, the major complication is embolization of the thrombus to the lung (4). 

Extensive epidemiological studies of patients with thromboembolism have identified several factors that enhance the risk of DVT development. These include surgery, age, gender, heart failure, history of previous thromboembolism, direct trauma to the leg, use of oral contraceptives, and limb weakness (3, 5). The majority of intensive care unit (ICU) patients have one or more risk factors for DVT (3). These patients are further predisposed to DVT during their ICU stay due to prolonged immobilization, sepsis, and vascular injury from indwelling central venous catheters or other invasive interventions (6). 

The incidence of reported DVT is likely to vary across populations, based on baseline characteristics of patients, post-ICU admission events and patient condition, clinical suspicion for VTE, the scheduling and methods of VTE screening, and prophylactic interventions. A retrospective study on ICU patients undergoing color-flow Doppler sonography for DVT screening has shown an estimated incidence of 33% (5). Cross-sectional studies of medical and surgical ICU patients have shown that approximately 10% have proximal DVT on admission to the ICU (7, 8). 

One study reports a VTE rate of 9 per 10,000 hospital admissions in an Iranian population (9). Based on the above mentioned, the aim of the present study was to determine the incidence of DVT among medical and surgical ICU patients. 

## Methods

***Study design and setting***

In this retrospective cross sectional study, patients older than 18 years who were hospitalized in ICU of Imam Hossein educational Hospital, Tehran, Iran, for ≥ 2 days were evaluated. The study was conducted in accordance with the principles of the 18^th^ world medical assembly (Helsinki, 1964) and all subsequent amendments. The study received ethical approval from Shahid Beheshti University of Medical Sciences.

***Participants***

All consecutive patients admitted to the ICU for the first time, between August 2008 and July 2011, were considered for inclusion in the study. Patients were excluded if they either had a documented DVT/PE before or within 48 hours of ICU admission or died or had missing or incomplete data during that time period. In this 21 bed ICU, a large variety of medical, surgical, orthopedics, neurosurgical, trauma, cardiac surgery and obstetrics and gynecology patients are managed. The ICU care is directed and run by trained intensivists in a semi-closed fashion with an average patient length of stay of 8 days.

***Data collection***

We collected data from patients’ electronic medical records and a regional ICU database. For each patient with clinically and Doppler proven DVT, key demographic and clinical characteristics including age, sex, diagnosis on admission, Acute Physiology and Chronic Health Evaluation II (APACHE II) score, VTE prophylaxis regimen, duration of mechanical ventilation, length of ICU stay and patient’s outcome (discharge or death) were collected. To provide a uniform and unbiased assessment of Doppler proven DVT, one research associate, who was a vascular surgeon, performed the Doppler examination for all patients potentially having DVT and was blinded to the patients’ history and clinical status. All clinical decisions were made at the discretion of the ICU team. Potentially having DVT was defined as International Classiﬁcation of Diseases, 9th Revision, Clinical Modiﬁcation (ICD-9-CM) codes (453.40, 453.41, 453.42, 453.80, and 453.90). 

***Statistical Analysis***

All statistical analyses were performed using SPSS version 19. Continuous data were expressed as mean and standard deviation for normally distributed data or median and inter-quartile range for non-normally distributed data. Categorical data were summarized as counts and percentages. Continuous data were contrasted between groups using the t-test or rank test as appropriate. Categorical data were contrasted using the chi-squared test. Multiple logistic regressions were used to identify independent risk factors associated with the presence of DVT. A p value less than 0.05 was considered statistically significant. 

## Results

Out of the 1387 reviewed patient files during the study period, 500 (36.04%) patients had been diagnosed as potentially having DVT and underwent venous color Doppler ultrasonography. Table 1 shows the baseline characteristics of patients. Based on the results of ultrasonography, 48 (3.5%) cases had DVT (62.5 % male). Their mean age was 60 ± 18 years and they had the mean APACHE II score of 16.3±5.1 and ICU mortality rate of 27.1%. Table 2 summarized characteristics of these patients. None of the patients had bleeding or treatment-related complications during the study. Univariate analysis showed significant correlation of age (p = 0.02) and length of ICU stay with incidence of DVT (p = 0.01) (Table 2). Based on multivariate analysis, only a longer ICU stay significantly associated with DVT incidence (OR: 1.07 per each day of ICU stay; 95% CI: 1.03–1.22; p value, 0.01).

## Discussion

The results of the present study revealed 3.5 % incidence of DVT in ICU admitted patients of Imam Hossein Hospital. DVT is difficult to diagnose because of the poor sensitivity and specificity of clinical symptoms and signs (10). DVT usually originates in the calf veins where the risk of subsequent complications is low. The classic symptoms and signs are due to venous obstruction and an inflammatory response in the affected area. However, the majority of DVT cases remain clinically silent (11).

The diagnostic gold standard tools for DVT and PE were venography and pulmonary angiography, both of which are invasive and costly. Doppler sonography examination has emerged as the noninvasive imaging method of choice for evaluation of DVT. It has the added ability of being able to visualize other pathologies mimicking venous obstruction (3).

The incidence of DVT in this survey is lower than that reported in Chinese (19%) and Caucasian (28- 32%) medical ICU patients not receiving prophylaxis (12-14).

Many factors could contribute to this relatively low incidence in comparison to other countries (15). There is an association between age and incidence of DVT (16). The Iranian population, like other Middle Eastern countries, is very young; while about 43% of the Western population are 40 years and above, only 18% of Iranians are in this age range (17). However, with increasing age of the Iranian population, DVT will probably become a growing public health problem. 

**Table 1:** Baseline characteristics of studied patients

**Parameters**	**N (%)**	**APACHE II**	**DVT n (%)**	**Incidence Rate** *
**Age (year)**				
≤40	492(35.47)	13.7 ± 4.7	10 (2.03)	1.64
46 - 65	391(28.19)	14.4 ± 5	16(4.09)	3.07
≥66	504(36.34)	18.9 ± 4.2	22(4.37)	3.04
**Sex**				
Male	807(58.18)	17 ± 5.4	30(3.72)	2.84
Female	580(41.82)	15.2 ± 4.5	18(3.10)	2.26
**ICU stay (day)**				
≤7	722(52.05)	15.6 ± 4.7	8(1.11)	2.45
8-28	500(36.05)	16.4 ± 5.9	24(4.80)	3.33
≥29	165(11.90)	16.5 ± 4.2	16(9.70)	1.99
**Year**				
2008	263(18.96)	13.1 ± 6.5	7(2.66)	1.92
2009	476(34.32)	16.6 ± 4.5	21(4.41)	3.08
2010	514(37.06)	16.1 ± 5.4	14(2.72)	2.15
2011	134(9.66)	19.5 ± 3	6(4.48)	3.83

* Rate per 1000 person days; APACHE II: Acute Physiology and Chronic Health Evaluation II; DVT: deep vein thrombosis; ICU: intensive care unit; data were presented as mean ± standard deviation or number and percentage.

**Table 2:** Characteristics of cases potentially having deep vein thrombosis (DVT) among intensive care unit (ICU) admitted patients

**Variable**	**Color Doppler ultrasound** **for DVT**		**P Value**
	**Negative (n=452)**	**Positive (n=48)**	
**Age (year)**	51 ± 19	60 ± 18	0.02
**Gender (male)**	259 (57.3%)	30 (62.5%)	0.6
**APACHE II**	14.2 ± 5.4	16.1 ± 4.8	0.2
**ICU stay (Day)**	21 ± 31	30 ± 30	0.01
**Diagnoses**			
Acute Hemorrhagic Stroke	82 (18.1%)	10 (20.8%)	0.7
Multiple Trauma	124 (27.5 %)	12 (25%)	0.8
Respiratory Distress	49 (10.8%)	6 (12.5%)	0.7
Acute Abdomen	45 (9.9%)	5 (10.4%)	0.8
Acute Ischemic Stroke	48 (10.6 %)	5 (10.4%)	0.9
Cancer	23 (5.1%)	3 (6.2%)	0.7
Sepsis	36 (8%)	3 (6.3%)	0.7
Loss of consciousness	24 (5.3%)	1 (2.1%)	0.6
Others	21(4.7%)	3 (6.2%)	0.7
**Type of DVT Prophylaxis**			
LMWH	137 (30.3%)	19 (39.6%)	0.5
Heparin	191 (42.2%)	12 (25%)	0.05
IPC and GCS	124 (27.5%)	17 (35.4%)	0.5
**ICU Mortality**	88 (19.5%)	13 (27.1%)	0.5

APACHE II: Acute Physiology and Chronic Health Evaluation II; LMWH: low molecular weight heparin; IPC: Intermittent Pneumatic Compression; GCS: graduated compression stockings; DVT: deep vein thrombosis, ICU: intensive care unit; data were presented as mean ± standard deviation or number and percentage.

On the other hand, it is likely that increased physician awareness of DVT risk factors and increased utilization of noninvasive imaging have led to a higher diagnosis rate (18). 

DVT incidence increases rapidly with age in an apparently linear form suggesting a constant incidence and cumulative prevalence, especially in those older than 65 years (1). Old age is one of the risk factors in patients with DVT (16).

The mean age of patients was almost 60 years, which was within the age range of people prone to venous thromboembolism (over 40 years of age) (1). In addition, consistent with previous studies, which have shown DVT to be more common among men (9), approximately 62.5% of our DVT patients were male. 

However, length of ICU stay was the most significant factor associated with the presence of DVT. The interpretation is that in many patients, DVT developed after they were admitted to the ICU and the risk of DVT increased with a longer ICU stay. 

The mean APACHE II score was 16.3 ± 5.1 and ICU mortality rate was 27.1% (13 in the 48 patients with DVT). The in-hospital mortality rate, observed in this study, appears similar to those reported from other studies in major academic health centers (2, 19). It is not clear whether this increase in fatality should be attributed to the accompanying risk factors, or to the severity of DVT (proximal), most likely both play an important role (20). We suggest that prophylaxis be administered to those with three or more risk factors. The mortality rate of DVT has a strong association with other risk factors such as, cardiovascular disease, cancer, states following surgery or trauma (2, 21). 

Evidence-based consensus guidelines for VTE prophylaxis have been available since a long time ago (22). The American College of chest physicians (ACCP) guidelines recommend prophylaxis for patients at moderate-to-high risk of VTE, using mechanical prophylaxis and/or pharmacological prophylaxis (19, 23). Despite the recommendations of international guidelines, physicians often do not prescribe prophylaxis therapy in high-risk situations (24). 

Means to improve DVT prophylaxis should include increasing physicians’ awareness through training and the implementation of procedures to assess DVT risk during hospitalization, along with the application of evidence-based guidelines for DVT prophylaxis and treatment in both medical and surgical patients (23). 

There are no local guidelines for DVT prevention in Iran. Considering that DVT is a serious clinical condition that may lead to patient death, we recommend the assessment of DVT risk (using a risk model assessment form, locally adapted) for all hospitalized patients on admission and during hospitalization. 

## Limitations:

The main limitation of our study is its retrospective and single center design. Prospective multicenter studies are recommended.

## Conclusion

The results of this study showed the 3.5% incidence of DVT in ICU admitted patients of Imam Hossein Hospital, Tehran, Iran. Longer ICU stay and older age were independent risk factors of DVT development. 
